# Hyaluronan production and chondrogenic properties of primary human chondrocyte on gelatin based hematostatic spongostan scaffold

**DOI:** 10.1186/1749-799X-7-40

**Published:** 2012-12-19

**Authors:** Jeerawan Klangjorhor, Puwapong Nimkingratana, Jongkolnee Settakorn, Dumnoensun Pruksakorn, Taninnit Leerapun, Olarn Arpornchayanon, Sattaya Rojanasthien, Prachya Kongtawelert, Peraphan Pothacharoen

**Affiliations:** 1 Thailand Excellence Center for Tissue Engineering and Stem Cells, Faculty of Medicine, Chiang Mai University, Intravarorot Road, Sripoom, Chiang Mai, 50200, Thailand; 2 Musculoskeletal Research Laboratory, Department of Orthopedics, Faculty of Medicine, Chiang Mai University, Intravarorot Road, Sripoom, Chiang Mai, 50200, Thailand; 3 Department of Pathology, Faculty of Medicine, Chiang Mai University, Intravarorot Road, Sripoom, Chiang Mai, 50200, Thailand

**Keywords:** Human articular chondrocyte 3D culture, Hyaluronan, Collagen, Gelatin, Spongostan, Scaffold

## Abstract

**Background:**

Autologous chondrocyte transplantation is a promising technique for treatment of cartilage defects. Three dimensional chondrocyte cultures on a scaffold are widely used to retain the chondrogenic phenotype. Using a biodegradable gelatin scaffold is one option for the cell delivery system, but molecular and histological studies of the method have not yet been done.

**Methods:**

We evaluated the chondrogenic property of the primary human chondrocyte on a gelatin scaffold as compared to a collagen scaffold over a period of 21 days. We examined the production of glycosaminoglycan by quantitative and histological analysis. Gene expression of cartilage-associated molecules was assessed by quantitative RT-PCR.

**Results:**

The gelatin scaffold showed the ability to promote chondrocyte expansion, chondrogenic phenotype retention at molecular and mRNA levels.

**Conclusions:**

This scaffold is thus suitable for use as an *in vitro* model for chondrocyte 3D culture.

## Introduction

Articular chondrocytes have limited ability to self-repair, and usually dedifferentiate to fibroblasts when grown in a monolayer culture. A three–dimensional (3D) culture system can be used to retain the chondrogenic phenotype. Matrix induced autologous chondrocyte implantation (MACI), a third generation ACI technique, was introduced in 1998 and developed as a treatment for chondral and subchondral defects of joint surfaces [1,2]. During the 5 years following its introduction, the technique underwent clinical improvements and proved suitable for treatment of cartilage defects [1]. A biodegradable scaffold is needed as a vehicle to deliver the precultured chondrocytes to the cartilage defects. The biomaterial scaffolds provide a suitable environment for chondrocytes to maintain their chondrogenic phenotype. Various types of natural substance scaffolds include fibrin, agarose [3], alginate [4], collagen [5,6], chitosan [7] and hyaluronan [8]. In addition supplementary methods include conditional use of 3D culture, hormones [9], growth factors [10], variation of oxygen tension [11], mechanical stimulation [12] and retrovirally transduced SOX9 genes in the chondrocytes [13]. The ideal scaffold should promote the chondrocytes to produce extracellular matrix, which constitutes the regenerated cartilage. It has previously been shown that a gelatin-based scaffold (Spongostan^TM^, Johnson & Johnson, Norderstedt, Germany) may be used as a 3D chondrocyte matrix [14]. Most studies have reported the total extracellular matrix production rather than glycosaminoglycan concentration. In this study we determined whether human articular chondrocytes embedded in a gelatin based scaffold could retain the chondrogenic properties required for chondrocyte transplantation, and compared this to collagen based scaffolds. We then performed quantitative biochemical and histomorphological analysis over a period of 21 days.

## Materials and methods

### Cell culture and reagents

Human chondrocytes from non-osteoarthritis joints were harvested from notchpalsty operation from articular knee cartilage of 18–45 years old patients and chondrocytes were isolated and purified by standard protocol. All patients gave consent and all procedures of this study were approved by the Ethical Committee of Chiang Mai University (approval no. 070CT111016). The gelatin scaffold was obtained from Johnson & Johnson (Germany). The collagen scaffold was purchased from BD Bioscience.

All materials and reagents used in this study were purchased from Sigma (Sigma –Aldrich USA) unless stated otherwise.

### Cell seeding and culture of scaffold

The primary human chondrocytes were grown and expanded as a monolayer in 10% (v/v) FCS DMEM (Gibco, Germany) containing 10 U/ml penicillin and 100 μg/ml streptomycin up to passage 4 (P4) for use in these experiments. Scaffold cultures were performed by directly seeding the chondrocytes (5×10^5^ cells suspension in 30 μl culture medium), onto the scaffold and leaved the chondrocytes suspension adsorbed and embedded in scaffold for 4 hrs. After that added chondrogenic media compose of DMEM supplemented with 10% FBS, 1X ITS +1, 25 μg/ml ascorbic acid 2-phosphate, 10^-7^ M dexamethasone, 10 ng/ml TGF-β and 100 ng/ml IGF-1 and allowed to adhere in a humidified incubator (37°C, 5% CO_2_) for 24 hrs. The chondrocytes embedded in the scaffolds were transferred to new culture wells. The numbers of non-adhering cells were measured by trypsinization of the culture well adhering cells and counted by a hemacytometer.

### Extracellular matrix (ECM) biomolecules assay

#### s-GAG assay

The sulfated glycosaminoglycan (s-GAG) was determined using the dimethylmethylene blue (DMMB) assay [15] with chondroitin 6-sulfate (CS-C: 0–60 μg/ml) as a standard. The DMMB solution was added to the sample and standards prior to reading absorbance values at 525 nm in a microplate reader spectrophotometer.

#### HA assay

Hyaluronic acid was measured using a competitive based ELISA as previously described [16]. Briefly, samples or standard HA (Healon®) at various concentrations (19–10,000 ng/ml) in PBS pH 7.4, were added to 1.5 ml plastic tubes containing biotinylated HABPs (1:200 in 0.05 M Tris–HCl buffer, pH 8.6). The tubes were incubated at room temperature for 1 hr. and then samples were added to the microplate, which was pre-coated with umbilical cord HA (100 μl/well of 10 mg/ml), and blocked with 1% BSA (150 μl/well). The plate was then incubated at room temperature for 1 hr. The wells were subsequently washed and 100 μl of peroxidase-conjugated anti-biotin antibody (1:2,000 dilution in PBS) were added. The plate was incubated at room temperature for another hour. The reaction was stopped after 10 minutes with 50 μl/well of 4 M sulfuric acid and the absorbance was determined using a microplate reader at 492/690 nm. The concentration of HA in samples was calculated by reference to a standard curve.

### DNA content

The total amount of DNA was measured by the Hoecht 33258 fluorescent dye assay. A 20 μl aliquot was diluted in 2 ml of dye/buffer solution and the fluorescence of the samples was evaluated for excitation at 450 nm and emission at 555 nm by a spectrophotometer. A standard curve was prepared from known concentrations of herring sperm.

### RNA extraction and gene expression analysis

Total RNA was isolated from scaffold culture using an RNA extraction kit (Amershame Bioscience) following the manufacturer’s protocol and the reagents in all extraction steps are RNases free solution. Total RNA (1 μg) was converted to cDNA using a RevertAid^*TM*^ First Strand cDNA synthesis kit (MBI Fermentas, Germany). For determination of gene expression, SYBR Green detection was used and the values were normalized using glyceraldehyde-3-phosphate dehydrogenase (GAPDH). Real-time quantitative polymerase chain reaction (PCR) was performed in a DNA Engine (ABi 7500) using SYBR GREENER qPCR UNIVERSAL (Invitrogen, USA). Primer sequences (from Invitrogen) are as follows:

Aggrecan core protein Forward 5’-3’ CTGTTCAGGGACAGAATGTGCT; Reverse 5’-3’ TCGATATGCTTCACAGTTCTAGGG: Collagen type I Forward 5’-3’ TTTTGGCCATCTCTTCCTTCA; Reverse 5’-3’ TGTGGATGCCTCTTGGGTATC: Collagen type II Forward 5’-3’ TCCTCTTCTTGAGCTGGACTCATTCT; Reverse 5’-3’ CGCTCTGCAAACTGGAGGTC: SOX9 Forward 5’-3’ GAAGGTGAAGGTCGGAGTC; Reverse 5’-3’ GAAGATGGTGATGGGATTTC. GAPDH Forward 5’-3’ GAAGGTGAAGGTCGGAGTC; Reverse 5’-3’ GAAGATGGTGATGGGATTTC. Relative expression levels for each primer set were normalized to the expression of GAPDH by the 2^*-ΔCT*^ method [17].

### Histology

For light microscopy, the samples were fixed in a 0.1 M phosphate-buffer (pH7.4) solution of 1% (v/v) paraformaldehyde and 1.25% (v/v) glutaraldehyde, dehydrated in alcohol and embedded in polymethylmethacrylate. Sections (4 μm) were stained with haematoxylin-eosin (H&E) and Safranin O.

For scanning electron microscopy, the samples were prefixed for 24 hrs. at 4°C with 2% (v/v) glutaraldehyde and 2% (v/v) tannic acid in 0.1 M sodium-cacodylate buffer (pH 7.4). Matrices were dehydrated through a graded series of ethanol and acetone; critical point dried using a CPD 7501 critical point Drier, mounted on stubs and spattered with an ultrathin layer of gold in a SEM coating system using SPI-MODULE^TM^ Spatter Coater. Specimens were studied using a JEOL 5910 LV SEM apparatus at an accelerating voltage of 15 kV.

### Statistical analysis

Data shown are representative of repeated experiments. Triplicate samples were used for chemical and biochemical assay. Duplicate samples were used for microscopy analysis. The data are expressed as mean ± SD. The significance of differences between groups was tested using non-parametric Wilcoxan rank-sum tests. Statistical significance was considered as *p <* 0.05.

## Results

### Production of glycosaminoglycan molecules

**Figure 1:**
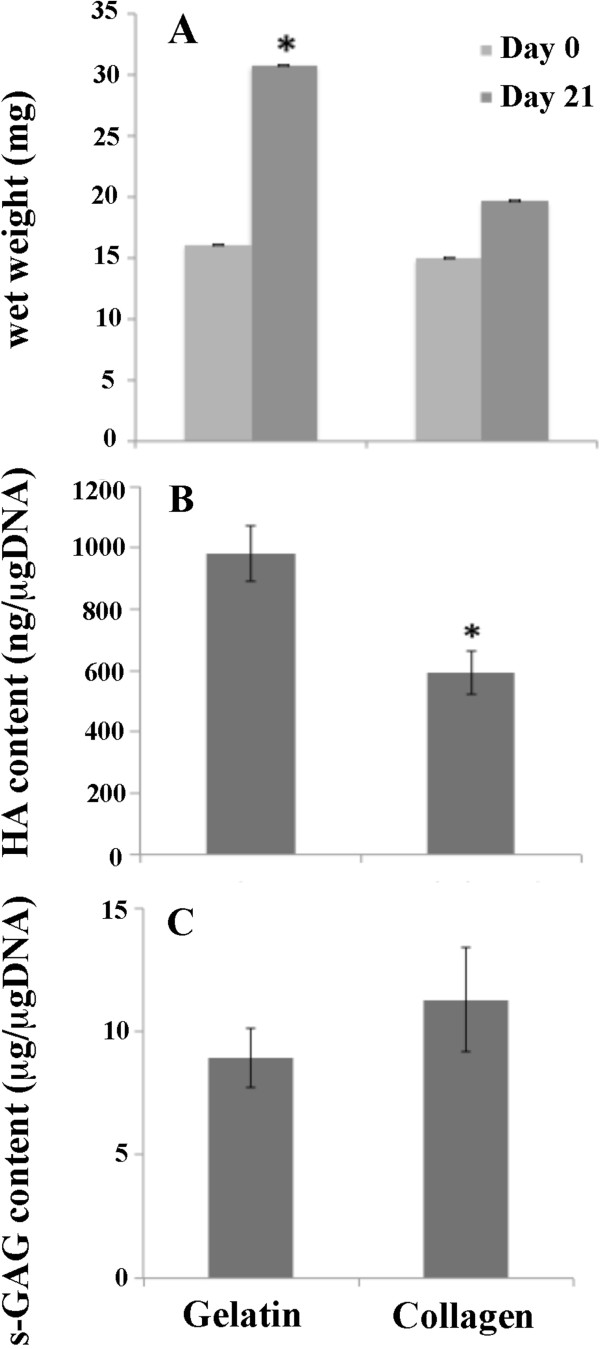
**Matrix glycosaminoglycan accumulation in scaffold.** Human articular chondrocytes (5x10^5^ cells) were cultured in each scaffold for 21 days. The wet weight (**A**), glycosaminoglycans content normalized to DNA content: HA (**B**), s-GAGs (**C**) were measured. Asterisk indicates significant (* p < 0.05).

**Figure 2:**
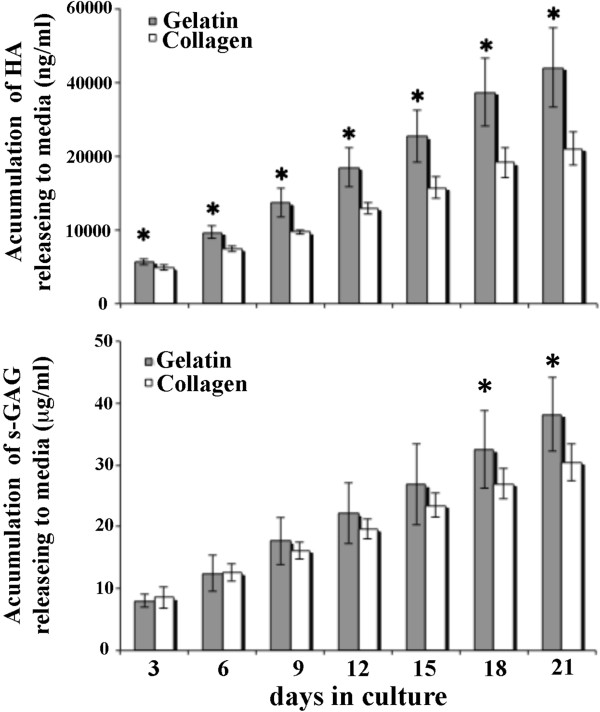
**The accumulative release of glycosaminoglycan: s-glycosaminoglycan (s-GAG) and hyaluronic acid (HA) from the gelatin and collagen scaffold into the medium during culture.** Asterisk indicates significant (* *p* < 0.05) increase of glycosaminoglycans production at the same time point.

The numbers of non-adhering chondrocytes in the gelatin were significantly higher than in the collagen scaffold (5,960 ± 1,113 *vs* 3,600 ± 285 cells: *p* < 0.05). The wet weight of the scaffolds was compared to assess extracellular matrix production. After 21 days, the gelatin scaffold had 1.5 fold increased in wet weight compared to the collagen scaffold (Figure 1A). We next examined the glycosaminoglycan content produced from the chondrocytes cultivated in the scaffold (Figure 2). For each 3 day-time point, the chondrocyte seeded gelatin scaffold showed higher levels of s-GAG (*p <* 0.05 at day 18 and 21) and HA production (*p* < 0.05 for all intervals) released into the media than the chondrocyte seeded collagen scaffold. After 21 days, the amount of HA/DNA content in the matrix in the gelatin scaffold was significantly higher than in the collagen scaffold (*p* < 0.05), although the GAG/DNA content in the matrix of the gelatin scaffold was slightly lower although not significantly less (*p* = 0.105) (Figure 1B, C).

### The chondrogenic mRNA profile

**Figure 3:**
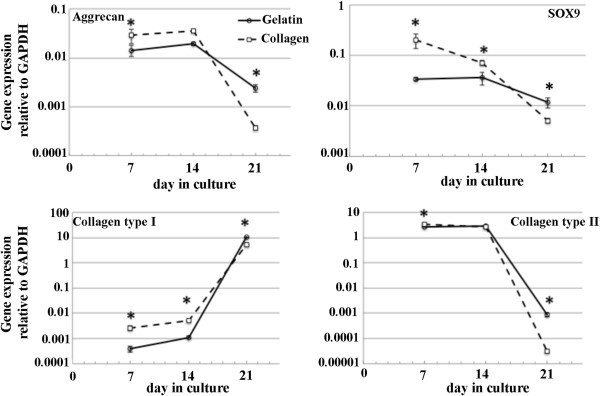
**Quantitative RT-PCR analysis of chondrocyte growth on gelatin and collagen scaffold over 21 days cultivation.** The results represent the chondrogenic gene expression (aggrecan core protein, SOX9, collagen type II and collagen type I). Values are expressed as the mean and standard deviation for 3 independent cultures. Asterisk indicates significant (*p < 0.05) increase of gene expression at the same time point.

Chondrogenic gene expression included Aggrecan core protein, Collagen type I Collagen type II and SOX9 were measured as shown in Figure 3. The mRNA analysis was performed at day 7 of the 21 days culture. On day 7, compared to the collagen scaffold, the expression of selected genes of the chondrocyte seeded gelatin scaffold were all significantly lower. After 14 days of cultivation, Aggrecan core protein and Collagen type II gene expression were unchanged from day 7, and significantly decreased at day 21 in both gelatin and collagen scaffolds. On day 21, the Aggrecan core protein and Collagen type II genes of the chondrocyte seeded gelatin scaffold had significantly higher expression levels compared to the collagen scaffold (*p <* 0.05). In contrast to Aggrecan and Collagen type II gene expression, Collagen type I gene expression was significantly different between the chondrocyte seeded gelatin and collagen scaffolds. In the both scaffold after cultivation for 14 days, Collagen type I gene expression was not significantly different from day 7, and was significantly increased at day 21. The chondrocyte seeded collagen scaffold showed a decrease of SOX9 gene expression after the 7 day interval while SOX9 expression of the chondrocytes that were seeded on the gelatin scaffold was unchanged from day 7, and on day 21 was decreased in comparison with day 14. Similar to Aggrecan core protein and Collagen type II, on day 21, the SOX9 gene expression of chondrocytes seeded on the gelatin scaffold was significantly higher compared with chondrocytes seeded on the collagen scaffold.

### Scanning electron microscopy

**Figure 4:**
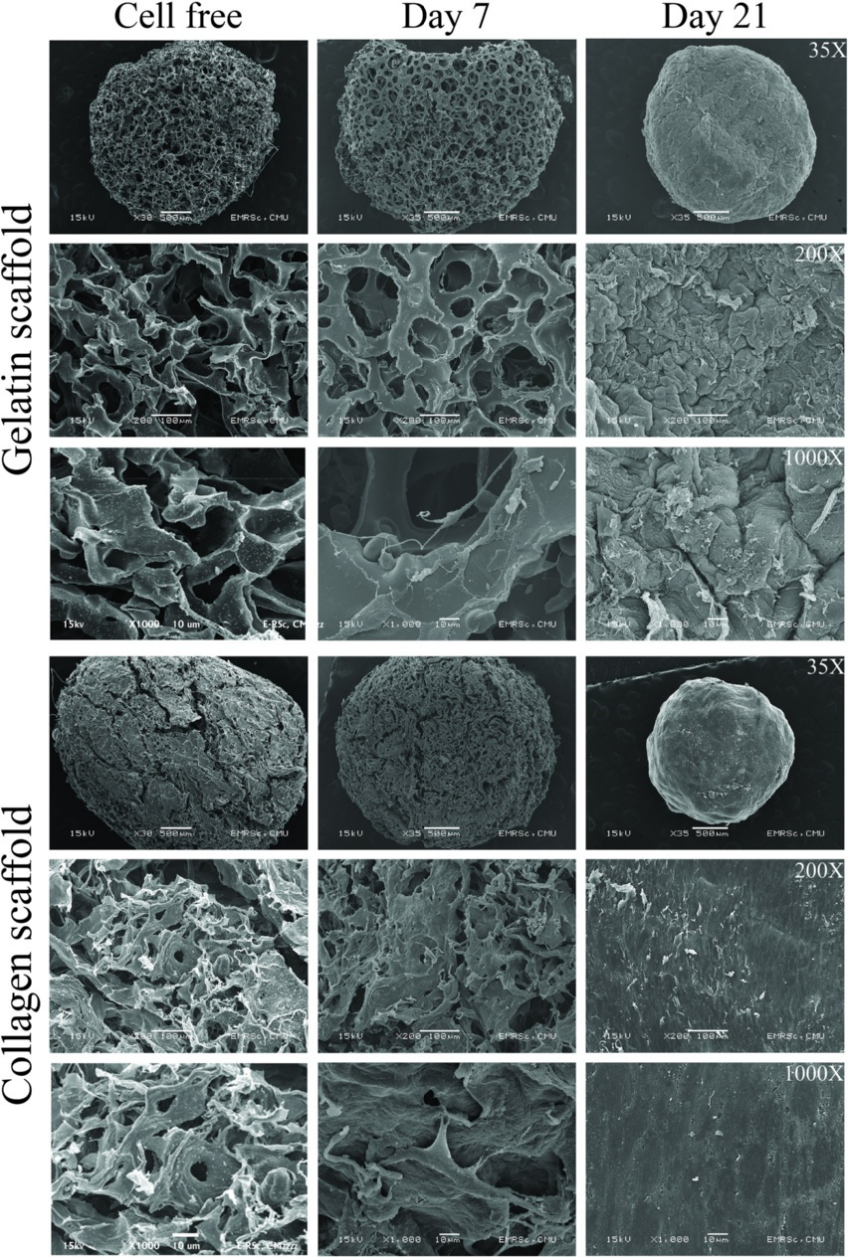
**Scanning electron microscope of the surface of gelatin and collagen scaffold.** Representative surface images of cell free scaffold, 7 and 21 days after chondrocyte seeding.

Cell morphology and cell distribution inside the scaffold was observed after 7 days culture by SEM. Matrix formation of the chondrocytes on the collagen scaffold was higher than on the gelatin scaffold, in which space-filled pores can be observed (Figure 4). Chondrocytes on both gelatin and collagen scaffolds showed a spherical morphology in agreement with previous studies [18]. The surfaces of the porous scaffolds are filled with the chondrocytes which are embedded in extracellular matrix and completely filling the pores of the scaffold on day 21. The surface of the chondrocytes on collagen scaffold was smoother than the chondrocytes on gelatin scaffold.

### Histology

**Figure 5:**
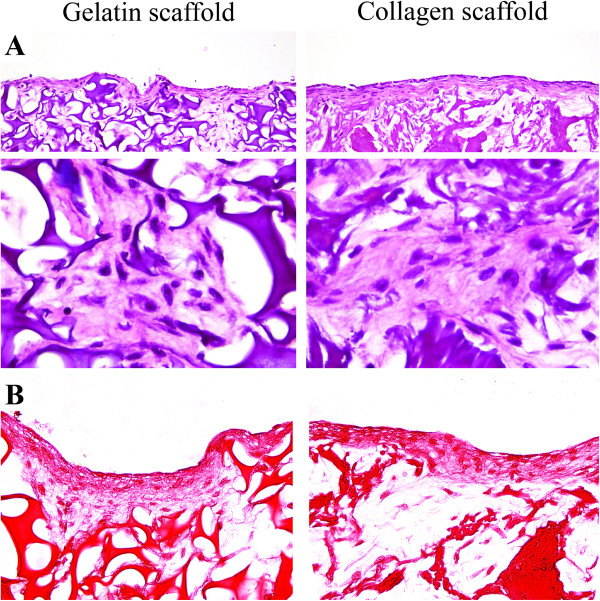
**Histological analysis of chondrocyte seeded gelatin and collagen scaffold.** (**A**) Hematoxylin&Eosin and (**B**) Safranin O stain of chondrocyte seed gelatin and collagen scaffold after 21 days in cultivation.

To determine cell penetration and morphology, hematoxylin eosin staining showed an equal distribution of chondrocytes in the porous material of both gelatin and collagen scaffolds (Figure 5A). The sulfated glycosaminoglycan production of the chondrocyte embedded scaffold was measured by Safranin O staining (Figure 5B). According to the GAG/DNA in the matrix (Figure 1C), the gelatin scaffold showed no difference in the intensity of Safranin O staining as compared to the collagen scaffold.

## Discussion

Gelatin, a porous form of denatured collagen, is used as a potential cell delivery system and to support bovine chondrocyte metabolism [19]. It has been used as a scaffold for cartilage tissue engineering [20,21]. In addition, gelatin scaffold (Spongostan^TM^ Johnson & Johnson) has suitable properties as a 3D chondrocyte matrix for bovine chondrocytes [14]. A collagen scaffold, with its inherent cartilage biological matrix, has been demonstrated to be a cell supporter and to enhance chondrogenic culturing of chondrocytes [12,22-24]. Based on the fact that the biological composition of gelatin is derived from collagen, a collagen scaffold should match its physio-biochemical properties, *in vivo* biodegradable property, and on the evidence from chondrocyte culture, we selected collagen scaffolds for comparison. We evaluated gelatin scaffolds (Spongostan^TM^ Johnson & Johnson) as chondrocyte 3D cultures for *in vitro* hyaline cartilage regeneration using human articular chondrocytes. The biochemical and histological analyses were designed to examine the influence of gelatin and collagen scaffolds on chondrocyte ECM synthesis over a 21-day culture period. Initially, we counted the numbers of non-adhering chondrocytes for estimate amount of chondrocytes which adhered into the scaffold to evaluate adhesive activity of chondrocyte to the scaffold and we found the level of chondrocytes adhering to the gelatin scaffold was significant less than in the collagen scaffold, due to the larger pore size (100–300 μm *vs* 100–200 μm). Gelatin is derived from denatured collagen, which retains an RGD motif peptide for cell adhesion and penetration. Even during initial cell adhering, there was evidence that chondrocyte seeded gelatin scaffolds had higher cell proliferation and chondrocyte matrix production, as shown by the DNA content and wet weight of the chondrocyte seeded gelatin scaffold being significantly higher than for the collagen scaffold after 21 days of culture.

The sulfated glycosaminoglycan production was not significantly different for both releasing and matrix forms. This was similar to the results from s-GAG staining among the two types of scaffold, which had no significant difference in matrix Safranin O stain intensity. Interestingly, the human chondrocytes seeded on the gelatin scaffold exhibited the higher HA production, for both matrix component and released form, than cells seeded on the collagen scaffold. The HA can serve as the core molecule for HA binding domains and linking proteins of cartilage matrix proteoglycans [25]. Moreover, via binding to cell surface receptors, this molecule plays a role in cell proliferation, morphogenesis and wound repair [26].

Our study examined quantitative chondrogenic gene expression at 7 day of a 21 days culture. At 7 days cultivation, the chondrocytes seeded collagen scaffold expressed higher levels of hyaline cartilage specific genes (Aggrecan core protein, Collagen type II and SOX9) than the gelatin scaffold. At day 14, a decrease in transcription factor SOX9 gene expression was observed. This protein is crucial for induction and regulation of the chondrocyte phenotype [27]. Aggrecan [28] and Collagen type II [29] expression are regulated by this transcription factor. A decrease in SOX9 induction affects the Aggrecan and Collagen type II expression. Thus, higher SOX9 expression on day 21 of the chondrocytes seeded gelatin scaffold potentially promoted the higher levels of Aggrecan and Collagen type II gene expression compared to the chondrocyte seeded collagen scaffold.

Our study described for the first time the preparation and characterization of primary human chondrocytes cultured in absorbable haemostatic gelatin (Spongostan^TM^) scaffold in extracellular matrix production including HA and GAG and specific chondrocyte differentiation gene markers. This observation indicated that the gelatin scaffold retained the chondrogenic response including the cells had not de-differentiation to the fibroblasts, enhanced extracellular matrix (HA and GAG) production and more potential to stabilized the expressions or mRNA levels of chondrogenic differentiation gene markers including SOX9 transcription factor, Aggrecan core protein and Collagen type II mRNA in late period of cultivation. Moreover in Collagen type I, dedifferentiation marker level was lower than chondrocytes cultured in collagen scaffold suggested that gelatin scaffold reduced de-differention of the cells. The phenotype maintaining ability of chondrocytes seeded on a scaffold is important for repairing cartilage defects. To our knowledge, this is the first study to report the quantification of HA production of a chondrocyte seeded scaffold. The higher HA production of the chondrocyte seeded gelatin scaffold over 21 days promoted cell proliferation and matrix synthesis and expression of cartilage specific matrix proteins.

In conclusion, our investigation has shown that a gelatin scaffold is adequate to support chondrocytes, and to induce extracellular matrix production of both HA and s-GAG. The high production of matrix HA from the chondrocyte seeded gelatin scaffold may prevent dedifferentiation via transmembrane receptor CD 44, resulting in promotion of cell proliferation and enhanced matrix molecule production. In addition, this scaffold supported expression of many cartilage matrix genes, including SOX9, Aggrecan and Collagen type II. Since a gelatin scaffold is a cheaper medical device, and showed the ability to support chondrocyte expansion and retention of the chondrogenic phenotype, it should be suitable as an alternative for use in *in vitro* 3D human chondrocyte culture as a regenerative cartilage model.

## Competing interests

The authors declare that they have no competing interests.

## Authors’ contributions

PP and JK performed most of the experiments, data analysis and manuscript preparation. PN, JS and DP performed Histological analysis. TL, SR, OA performed cartilage samples collection. PK conceived and developed the study design and participated in manuscript preparation. All authors read and approved the final manuscript.
